# Physics-guided networks for probabilistic hydrodynamic forecasting in canal systems

**DOI:** 10.1016/j.ese.2026.100703

**Published:** 2026-05-05

**Authors:** Wangjiayi Liu, Guanghua Guan, Xiaonan Chen, Liangsheng Shi, Guangtao Fu, Dragan Savic

**Affiliations:** a State Key Laboratory of Water Resources Engineering and Management, Wuhan University, Wuhan, 430072, China; b Construction and Administration Bureau of the Middle-Route of the South-to-North Water Division Project of China, Beijing, 100038, China; c Centre for Water Systems, University of Exeter, Exeter, EX4 4QF, United Kingdom; d KWR Water Research Institute, Groningenhaven 7, Nieuwegein, 3430 BB, Netherlands

**Keywords:** Uncertainty quantification, Physics-guided neural network, Deep probabilistic learning, Hydrodynamic model, Canal system

## Abstract

Reliable prediction of water supply dynamics in large-scale canal systems is critical for water allocation and operational decision-making in inter-basin water transfer projects. Uncertainty in lateral offtake discharges evolves over time and often exhibits multi-peaked distributions due to real-time hydraulic states and unplanned gate operations. However, reliably quantifying and interpreting the evolving uncertainty remains difficult under such dynamically changing and small-sample conditions. Here we show that a physics-guided mixture density network (PgMDN) can effectively characterize this uncertainty while remaining physically consistent. In the proposed PgMDN, physical knowledge is incorporated into the loss function through local mass balance and a consistency constraint between predictions and their associated uncertainty, while long short-term memory layers are employed to model temporal dependencies and multi-factor influences. In addition, Shapley additive explanation analysis is used to identify the dominant hydraulic inputs contributing to predictive uncertainty. Tested on real-world canal datasets, the proposed PgMDN outperforms the standard mixture density network, achieving over a 25% reduction in both mean absolute error and root mean square error, together with improved reliability, as measured by the R-index (increasing from 0.45 to 0.82), and stronger generalization. The results further reveal that water level fluctuations and boundary inflow are key drivers of predictive uncertainty, supporting the physical interpretability of the proposed model. Overall, this study provides a scalable and interpretable tool for real-time modeling of environmental infrastructure and the operational management of large-scale water diversion systems.

## Highlights

• A physics-guided MDN quantifies uncertainty in canal offtake discharges for water supply.
• PgMDN embeds local mass balance and prediction-uncertainty constraints into the loss function.
• PgMDN improves reliability and generalization under small-sample canal discharge data.
• SHAP analysis identifies key hydraulic factors contributing to predictive uncertainty.
• The approach supports adaptive and sustainable water allocation for system resilience.

## Graphical abstract

**Figure:**
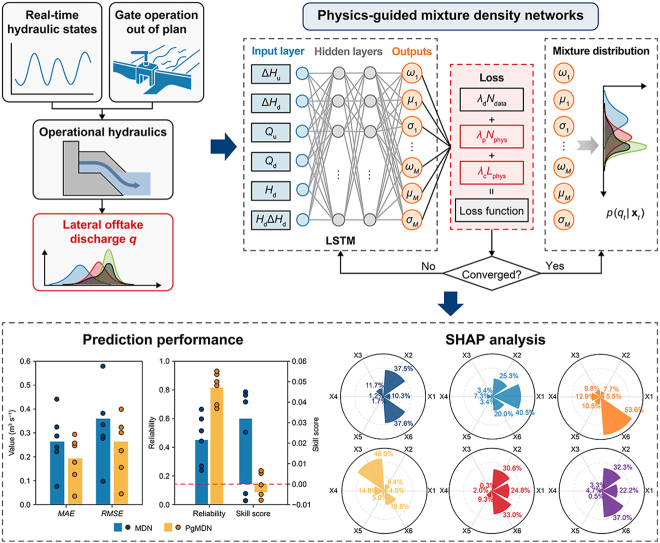

## 1 Introduction

Inter-basin water transfers play a vital role in redistributing water resources across spatial and temporal scales, supporting groundwater recovery, water security, and long-term ecological resilience under climatic and socio-economic change [[1], [2], [3]]. Reliable hydrodynamic predictions are essential for securing water supply and providing early risk warnings [4,5].

Compared with natural rivers or watershed systems, canal systems are influenced by both natural processes and human operations, with flow regimes shaped by gate operations and evolving hydraulic conditions. In such systems, lateral offtake discharges frequently deviate from planned supply targets. These deviations, driven by interactions with real-time system states and unplanned gate operations, produce multimodal distributions with multiple distinct peaks under given conditions [6,7]. This uncertainty degrades the accuracy of water-level predictions and may lead to ineffective operational decisions and delayed emergency responses [8]. Therefore, robust uncertainty quantification (UQ) of real-time water offtake discharges is urgently needed to support adaptive and resilient water distribution under dynamic and uncertain operational conditions.

The Monte Carlo (MC) method, a traditional numerical approach for UQ, relies on repeated random sampling to approximate the probability distributions of model predictions [9]. It is commonly applied to physics-based models, such as hydrologic simulations, by sampling inputs or parameters [10]. To improve efficiency when sampling from complex distributions, several acceleration strategies have been developed. Among these, Markov chain MC has been used to quantify prediction intervals for nonlinear flow behaviors [[11], [12], [13]], and Camacho et al. [14] demonstrated its effectiveness in both one- and multi-dimensional hydrodynamic modeling. Additionally, Latin hypercube sampling improves coverage of parameter spaces and reduces redundant simulations [15,16], while sequential MC enhances exploration efficiency in complex water systems [17].

Despite these advances, MC-based UQ requires carefully defined sampling ranges and remains computationally expensive. Moreover, such methods are ill-suited for predicting uncertainty in water-offtake discharges, as the absence of explicitly physical models makes it difficult to define input variables, often leading to unrealistic sampling.

As a more efficient alternative, statistical modeling approaches estimate predictive distributions directly from observed data. Parametric methods—assuming Gaussian or non-Gaussian distributions—have been widely applied in hydrodynamic modeling, including flood forecasting [18], stage-discharge relationships [19], and extreme precipitation analysis [20]. To increase flexibility, non-parametric methods that avoid strict distributional assumptions have also been explored. For example, data-driven classification approaches have been used to generate probabilistic flood forecasts from historical model errors [21], generalized likelihood uncertainty estimation has been linked with Bayesian inference in hydrological modeling [22], and bootstrapping techniques have been combined with hydrodynamic models for flood warning systems [23]. However, because these methods primarily rely on historical patterns and neglect dynamic interactions within the system, they struggle to capture the multimodal characteristics of future offtake discharges driven by real-time hydraulic conditions.

With the rapid adoption of deep learning for modeling complex, implicit input–output relationships, Bayesian neural networks (BNNs) and other stochastic, data-driven approaches have emerged as powerful tools for UQ [24,25]. Bayesian long short-term memory (LSTM) networks have been applied to simulate uncertainty in streamflow for data-scarce basins [26] and in soil moisture dynamics [27]. As an approximate Bayesian inference technique, MC dropout simulates an ensemble of networks by randomly deactivating neurons, enabling estimation of posterior distributions in hydrological models [28,29] and water distribution failure analysis [30]. To better represent real-world distributions, variational Bayesian inference has been used to model uncertainty in LSTM weights [31], while Gaussian mixture models have been integrated with LSTM networks for short-term forecasting [32].

McLachlan et al. [33] demonstrated that finite mixtures of Gaussian distributions can approximate any continuous probability distribution. Building on this principle, the mixture density network (MDN), originally proposed by Bishop [34], has been increasingly applied to improve UQ in hydrodynamic modeling. Due to their flexibility in representing multi-modal distributions, MDNs efficiently have been successfully used in applications such as stage-discharge curve estimation [35], hydraulic-geological modeling [36], and streamflow prediction [37,38]. Furthermore, comparative studies have shown that MDNs outperform MC dropout-based LSTM models in capturing complex and arbitrary distributions [39].

However, conventional MDN and BNN models often lack physical constraints and depend heavily on large, high-quality datasets, limiting their reliability in real-time hydrodynamic prediction. Although recent studies in other domains have incorporated physical knowledge into probabilistic neural networks to improve the reliability and interpretability [40,41], these approaches are typically designed for static tasks with single-variable inputs. In canal systems, uncertainty in offtake discharges arises from dynamic, multivariate hydraulic interactions, presenting additional challenges compared to natural hydrological systems. Consequently, existing approaches remain insufficient for modeling uncertainty in real-time offtake discharge prediction.

To address these limitations, this study proposes a physics-guided mixture density network (PgMDN) that integrates physical knowledge into probabilistic learning, enabling reliable real-time uncertainty modeling in canal systems. The proposed model captures dynamic, multivariate interactions while maintaining physically plausible predictions under data-limited conditions. In addition, the Shapley additive explanations (SHAP) analysis is employed to identify key hydraulic drivers of predictive uncertainty. The performance of the PgMDN is evaluated using real-world canal system data, demonstrating improvements in accuracy, reliability, and generalization.

## 2 Methodology

### 2.1 Uncertainty in the offtake discharge prediction

The hydrodynamic behavior of canal systems is governed by unsteady discharges through the upstream control gate (*Q*_u_), the downstream control gate (*Q*_d_), and lateral offtakes (*q*) [42]. To minimize water-level fluctuations caused by water withdrawal, offtakes are typically located near the downstream end of canal reaches [43]. In practice, *Q*_u_ and *Q*_d_ are often predetermined to meet water-supply or flood-control objectives. However, the offtake discharge *q* exhibits a multimodal distribution due to the influence of real-time hydraulic conditions and unplanned gate operations. Considering flow time travel delays and uncertainty in system information, the offtake discharge *q* in real-world systems can be expressed as follows:

(1) $$\tilde{q_{t}}=Q_{u,\left(t-t_{d}\right)}-Q_{d,t}+\varepsilon_{q,t}$$

where *t* is the time step; *t*_d_ represents the delay time step associated with water flow from the upstream end to the downstream end; *ε*_*q,t*_ denotes the residual error in the predictive offtake discharge at time step *t*; and $\tilde{q_{t}}$ is a stochastic variable (m^3^ s^−1^).

To quantify the inherent uncertainty in the predictive *q*, a cumulative distribution function (CDF) is used to characterize its probability distribution, obtained by integrating the corresponding probability density function. This probabilistic formulation enables representation of the stochastic and potentially multimodal nature of *q*. At each time step *t*, conditioned on a predictor vector (denoted ***x***_*t*_), the model outputs a conditional distribution *p*(*q*_*t*_|***x***_*t*_) with nonlinear, time-varying statistical characteristics (e.g., mean and variance).

### 2.2 Physics-guided mixture density network

#### 2.2.1 Standard MDN architecture

The MDN architecture employed in this study consists of four layers: one input layer, two hidden layers, and one output layer. Based on the availability of real-time sensor measurements, the input layer comprises water-level variations at the upstream and downstream (Δ*H*_u_ and Δ*H*_d_); planned discharges at the upstream and downstream (*Q*_u_ and *Q*_d_); the downstream water level (*H*_d_); and a nonlinear interaction term (*H*_d_Δ*H*_d_).

These variables collectively represent the hydraulic state of the system and the scheduled control actions, providing information relevant to subsequent offtake discharge. In particular, *H*_d_ and *H*_d_Δ*H*_d_ characterize downstream hydraulic conditions, which directly influence offtake flow, as offtakes are typically located near the downstream end. The selected variables enable the model to capture both natural and operational influences embedded in the hydraulic state.

Because water-level states are derived from past observations, whereas discharge plans are specified in advance, the predictor vector at time step *t* includes variables from different time steps. Specifically, Δ*H*_u_, Δ*H*_d_, *H*_d_, and *H*_d_Δ*H*_d_ are taken from the time step *t* − 1, whereas *Q*_u_ and *Q*_d_ correspond to time step *t*. Accordingly, the predictor vector ***x***_*t*_ is constructed using variables from both *t* and *t* – 1, as expressed in equation (2):

(2) $$x_{t}=\left\{\Delta H_{u,t-1},\Delta H_{d,t-1},Q_{u,t},Q_{d,t},H_{d,t-1},{\left(H_{d}\Delta H_{d}\right)\;}_{t-1}\right\}$$

where Δ*H*_u,*t*−1_ and Δ*H*_d,*t*−1_ denote changes in upstream and downstream water levels at time step *t* − 1 (m), respectively; *Q*_u,*t*_ and *Q*_d,*t*_ denote planned discharges through the upstream and downstream control gates at time step *t* (m^3^ s^−1^), respectively; and *H*_d,*t*−1_ denotes the downstream water level at time step *t* − 1 (m).

As a flexible probabilistic framework, the MDN incorporates two stacked LSTM layers to capture temporal dependencies in the input sequence. The output of the second LSTM layer is mapped to the parameters of the mixture distribution. Each LSTM processes a historical input sequence spanning *T* time steps (***x***_*t*−*T*_, …, ***x***_*t*_), and produces a multimodal predictive distribution for *q* at the *t*th time step (*q*_*t*_) in a sequence-to-one architecture. Key hyperparameters—including the number of neurons per layer, number of training epochs, learning rate, and batch size—are tuned during model development [39]. The MDN outputs an estimated CDF represented as a mixture of component distributions with different weights [44]. The conditional distribution *p*(*q*_*t*_|***x***_*t*_) can be expressed as equations (3), (4):

(3) $$\left(q_{t}|x_{t}\right)=\sum_{i=1}^{M}\omega_{i,t}\left(x_{t}\right)\phi \left(q_{t}|\theta_{i,t}\left(x_{t}\right)\right)$$

(4) $$\sum_{i=1}^{M}\omega_{i,t}\left(x_{t}\right)=1,0\leq \omega_{i,t}\left(x_{t}\right)\leq 1$$

where *M* denotes the number of mixture components and *ω*_*i,t*_(***x***_*t*_) is the mixing weight of the *i*th kernel *ϕ*(•) at time step *t*. The kernel function *ϕ*(•) can be any type of parametric density distribution and is governed by a vector of parameters *θ*_*i,t*_(***x***_*t*_) (e.g., mean *μ* and standard deviation *σ*).

In this study, the number of mixture components (*M*) is set to 3. The kernel function *ϕ*(•) follows a Gaussian distribution due to its flexibility and effectiveness in probabilistic modeling [45]. *θ*_*i,t*_(*x*_*t*_) can be further expressed as (*μ*_*i,t*_, *σ*^*2*^_*i,t*_), where *μ*_*i,t*_(***x***_*t*_) and *σ*^*2*^_*i,t*_(***x***_*t*_) indicate the mean and variance of the *i*th Gaussian component, respectively. Accordingly, outputs of the MDN contain a set of time-varying parameters (*ω*_*i,t*_, *μ*_*i,t*_, *σ*^*2*^_*i,t*_). To ensure valid parameterization, activation functions are applied in the output layer: A Softmax function enforces that the mixture weights *ω*_*i,t*_ sum to 1, while a Softplus function ensures positivity of the variances *σ*^*2*^_*i,t*_; the means *μ*_*i,t*_ retain their original values [34]. Model training is performed using the negative log-likelihood (NLL) function based on the observed data:

(5) $$N_{\text{data}}=-\sum_{i=1}^{N}\;\ln\left[\sum_{i=1}^{M}\omega_{i,t}\left(x_{t}\right)g\left(q_{o,t}|\mu_{i,t}\left(x_{t}\right),\sigma_{i,t}^{2}\left(x_{t}\right)\right)\right]$$

where *N*_data_ denotes the NLL term based on observed data; *g*(•) denotes the Gaussian distribution; *q*_o_ represents observed values of *q* in m^3^ s^−1^; and *N* is the total number of time steps.

The trained model is then used to predict the distribution parameters of the conditional distribution *p*(*q*_*t*_|***x***_*t*_). Based on these parameters, quantiles of the mixture distribution can be computed to quantify predictive uncertainty. The mixture-weighted mean (*μ*) represents the expected value of *q*, while the mixture-weighted variance (*σ*^2^) reflects uncertainty. Because the inverse CDF of the mixture distribution lacks a closed-form solution, the 90% prediction interval is approximated using MC sampling with 10,000 draws from the fitted Gaussian mixture.

#### 2.2.2 MDN with physical constraints

**Fig. 1:**
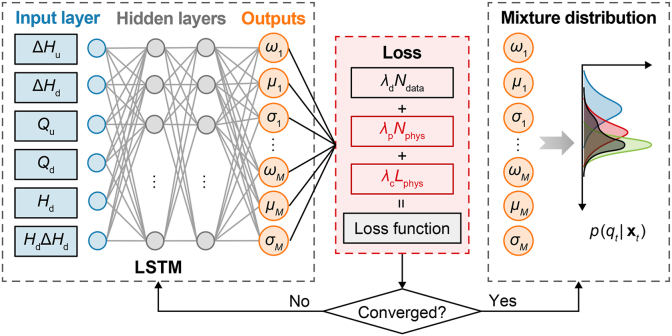
Architecture of the PgMDN, showing the input variables, LSTM–MDN structure, physics-guided loss function, and conditional output distribution. The network outputs the mixture weights, means, and standard deviations of the Gaussian components to parameterize the conditional probability distribution *p*(*q*_*t*_|***x***_*t*_). PgMDN, physics-guided mixture density network; LSTM, long short-term memory; MDN, mixture density network; Δ*H*_u_, upstream water-level variation; Δ*H*_d_, downstream water-level variation; *Q*_u_, upstream discharge; *Q*_d_, downstream discharge; *H*_d_, downstream water level; *H*_d_Δ*H*_d_, interaction term between downstream water level and downstream water-level variation; *ω*, mixture weight; *μ*, mean; *σ*, standard deviation; *M*, number of Gaussian components; *N*_data_, the NLL term based on observed data; *N*_phys_, the physical NLL term derived from local mass balance; NLL, negative log-likelihood; *L*_phys_, physics-guided consistency term between mean variation and predictive uncertainty; *λ*_d_, *λ*_p_, and *λ*_c_, the weighting coefficients of the data NLL term, the physical NLL term, and the physics-guided term, respectively.

To improve real-time prediction of offtake discharge under limited data conditions, we incorporate local flow continuity constraints and a coupling relationship between predictive mean variation and uncertainty into the MDN framework, resulting in the proposed PgMDN model. While retaining most components of the original MDN architecture, the PgMDN introduces two physics-based constraint terms into a unified loss function to guide model training. These additional terms influence the back-propagation process by adjusting internal network parameters during forward propagation, thereby enhancing the physical consistency of the model predictions (Fig. 1); further details are provided below.

(1) A physical NLL term based on the mass balance equation at the offtake

Unlike the standard MDN loss function, which minimizes only the discrepancy between predicted means and field-observed values, the proposed physical NLL term incorporates a governing physical equation as an additional training objective. Based on the mass balance principle, the predicted mean values of *q* from the PgMDN are encouraged to be consistent with the difference between inflow (*Q*_in_) and outflow (*Q*_out_) within the computational grid containing the offtake.

The values of *Q*_in_ and *Q*_out_ are generated using a physics-based model derived from one-dimensional (1D) hydrodynamics. This model is based on simplified hydraulic assumptions and is driven by observed data from a real-world canal system, including boundary discharges, initial water surface profile, and offtake discharges during the training period. Notably, observed water levels capture complex hydraulic effects present in real canal systems, including unmodeled losses, and are therefore used as key inputs for offtake discharge prediction. Based on these inputs, *Q*_in_ and *Q*_out_ are simulated by solving the 1D discrete Saint-Venant equations using the four-point Preissmann scheme [46].

This physical constraint is incorporated into the loss function via an NLL formulation similar to equation (5), with the observed-value term replaced by the mass-balance term. Rather than enforcing strict equality, this formulation promotes consistency between predicted mean values and underlying physical laws, as expressed in equation (6):

(6) $$N_{\text{phys}}=-\sum_{i=1}^{N}\;\ln\left[\sum_{i=1}^{M}\omega_{i,t}\left(x_{t}\right)g\left({\left(Q_{\text{in}}-Q_{\text{out}}\right)}_{t}|\mu_{i,t}\left(x_{t}\right),\sigma_{i,t}^{2}\left(x_{t}\right)\right)\right]$$

where *N*_phys_ denotes the physical NLL term derived from local mass balance; *Q*_in_ and *Q*_out_ denote the inflow and outflow discharges within the computational grid containing the offtake (m^3^ s^−1^), respectively.

Although this constraint is explicitly applied only to the predicted mean, it also influences the variance due to the internal coupling between mean and variance within the joint output layer of the PgMDN.

(2) A physics-guided term constraining the relationship between changes in predicted means (Δ*μ*) and the corresponding standard deviation (*σ*)

Significant variations in the predicted mean values of *q* often reflect rapid changes in the underlying hydraulic state of canal systems [6]. Such fluctuations are typically associated with dynamic transitions and potential unplanned or abrupt gate operations. Consequently, large changes in predicted mean values can be interpreted as indicators of reduced system stability, under which predictive uncertainty is expected to increase. Based on this physical insight, a positive correlation is assumed between the magnitude of changes in predicted discharge (*q*) and the corresponding uncertainty.

To incorporate this assumption into model training, a physics-guided term is added to the loss function to enforce consistency across predictions. This term penalizes the model when the evolution of predicted means and predictive uncertainty follows opposing trends. Specifically, increases (or decreases) in mean fluctuations should correspond to wider (or narrower) uncertainty intervals. This is implemented by encouraging directional consistency between changes in predicted means (Δ*μ*) and the standard deviation (*σ*), such that the loss approaches zero only when both vary in the same direction.

In addition, a parameter *α* is introduced to enable adaptive learning by imposing stronger constraints in regions that exhibit sharper variation during training. This mechanism allows the model to focus on dynamically unstable regions rather than uniformly inflating uncertainty and producing overly conservative predictions. The physics-guided term is defined in equations (7), (8), where *μ* and *σ* denote the mixture-weighted values of *μ*_*i*_ and *σ*_*i*_, respectively:

(7) $$L_{\text{ph}\text{ys}}=\sum_{t=2}^{N}\alpha_{t}{\left(E_{\Delta \mu ,t}\cdot E_{\sigma ,t}-\left|E_{\Delta \mu ,t}\right|\cdot \left|E_{\sigma ,t}\right|\right)}^{2}$$

(8) $$\alpha_{t}=\frac{e^{\left|E_{\Delta \mu ,t}\right|}}{\sum_{t=2}^{N}e^{\left|E_{\Delta \mu ,t}\right|}},E_{\Delta \mu ,t}=\Delta_{\mu ,t}-\Delta_{\mu ,t-1},\Delta_{\mu ,t}=\left|\mu_{t}-\mu_{t-1}\right|,E_{\sigma ,t}=\sigma_{t}-\sigma_{t-1}$$

where *L*_phys_ denotes physics-guided consistency term between mean variation and predictive uncertainty; *α*_*t*_ regulates the strength of the constraint; *e* denotes the base of the natural logarithm; *E*_Δ*μ,t*_ and *E*_*σ,t*_ represent the change trends of predicted mean variation and predicted variance, respectively; and Δ_*μ,t*_ denotes the absolute difference between adjacent predicted means.

The final loss function for PgMDN training is formulated as a sum of the data NLL term, the physical NLL term, and the physics-guided term, as shown in equation (9). In this study, all loss components are assigned equal weights to ensure comparable contributions during training. The back-propagation algorithm, therefore, seeks to minimize both data-fitting loss and physics-based constraint losses simultaneously.

(9) $$L=\lambda_{d}N_{\text{data}}+\lambda_{p}N_{\text{phys}}+\lambda_{c}L_{\text{phys}}$$

where *λ*_d_, *λ*_p_, and *λ*_c_ denote the weighting coefficients of the data NLL term, the physical NLL term, and the physics-guided term, respectively. In this study, *λ*_d_ = *λ*_p_ = *λ*_c_ = 1.

### 2.3 SHAP analysis

Identifying key contributors to model uncertainty is essential for understanding the behavior of the PgMDN and improving the credibility of its predictions. To this end, SHAP is employed to interpret the influence of each input variable on the predictive uncertainty interval, thereby enhancing the interpretability of the trained model [47].

Based on cooperative game theory, SHAP computes Shapley values to quantify each feature's average marginal contribution to model predictions [48]. This approach evaluates model behavior across all possible feature combinations, both including and excluding the feature of interest.

In this study, SHAP analysis is implemented for the mixture-weighted variance (*σ*^2^) predicted by the PgMDN. Shapley values quantify the relative importance of each input variable in influencing predictive uncertainty. By examining the consistency of feature importance across days, the dominant hydraulic factors governing uncertainty in water-offtake discharge can be identified.

### 2.4 Evaluation metrics

The MDN and PgMDN models are evaluated based on predictions of *q* from two perspectives: accuracy and uncertainty. Predictive accuracy is assessed using the mean absolute error (*MAE*) and the root mean square error (*RMSE*). In addition, the reliability index (*R*-index) and skill score (*S*-index) are used to evaluate model performance in uncertainty forecasting [49]. All metrics are computed across all prediction time steps, yielding a single evaluation value for each model.

*MAE* and *RMSE* quantify the average absolute and squared differences between predictions and observations, respectively, with lower values indicating better predictive performance. These metrics are defined in equations (10), (11):

(10) $$E_{MAE}=\frac{1}{N_{p}}\sum_{t=1}^{N_{p}}\left|q_{o,t}-q_{p,t}\right|$$

(11) $$E_{RMSE}=\sqrt{\frac{1}{N_{p}}\sum_{t=1}^{N_{p}}{\left(q_{o,t}-q_{p,t}\right)}^{2}}$$

where the *N*_p_ denotes the total number of prediction samples, and *q*_o_ and *q*_p_ represent observed and predicted values, respectively.

The *R*-index measures the proportion of observed data points that fall within the predicted uncertainty interval. A value closer to the predetermined confidence level indicates better agreement with the theoretical expectation; positive deviations are considered favorable, whereas negative deviations indicate undercoverage. Given sufficient reliability, a narrower uncertainty interval reflects improved predictive performance.

The *S*-index provides a comprehensive evaluation of both reliability and interval width, with values closer to zero indicating better overall performance. A positive *S*-index corresponds to confident (narrow) predictions, whereas a negative value indicates conservative (overly wide) intervals. Thus, values near zero reflect an optimal balance between reliability and sharpness [49].

Given a confidence level *τ*, the *R*-index *R*_*τ*_ and the *S*-index *S*_*τ*_ are defined as follows:

(12) $$\delta_{\tau }=\left\{ \begin{array}{c} 1,\text{if}\;q_{o,t}\leq \hat{q_{\tau ,t}}\\ 0,\text{otherwise}\; \end{array}\right.,R_{\tau }=\sum_{t=1}^{N_{p}}\frac{\delta_{\tau }}{N_{p}}$$

(13) $$S_{\tau }=\frac{1}{N_{p}}\sum_{t=1}^{N_{p}}\left(\delta_{\tau }-\tau \right)\left(q_{o,t}-{\hat{q}}_{\tau ,t}\right)$$

where *τ* is the prescribed confidence level (0.9 in this study); ${\hat{q}}_{\tau ,t}$ denotes the bounds of the uncertainty interval at a confidence level *τ* for time step *t* (m^3^ s^−1^); and *δ*_*τ*_ is a binary indicator specifying whether the observed value falls within the predicted interval.

## 3 Case study

In this study, small synthetic datasets generated by analytical functions are used to evaluate the MDN's baseline capability to learn diverse distribution patterns under limited data conditions. These datasets reflect representative statistical characteristics of real systems. In addition, real-world canal data are used to compare the MDN and PgMDN models, demonstrating the practical performance improvements achieved by the proposed approach.

### 3.1 Synthetic data

We generated three types of synthetic datasets.

(1) Case 1, a linear function with heteroscedastic Gaussian error;
(2) Case 2, a nonlinear function with a non-Gaussian, multimodal error distribution; and
(3) Case 3, a nonstationary time series with abrupt changes and multimodal noise.

The equations for Cases 1 and 2 are adopted from Li et al. [38], while Case 3 is constructed using piecewise functions incorporating regime switching and jump terms. Input variables are randomly generated within specified ranges: [0, 1] for Case 1 and [−1, 1] for Cases 2 and 3. The number of training and testing samples is set to match the number of samples in the real canal system data used for real-time prediction, as described in Section 3.3.

### 3.2 Real-world data

**Fig. 2:**
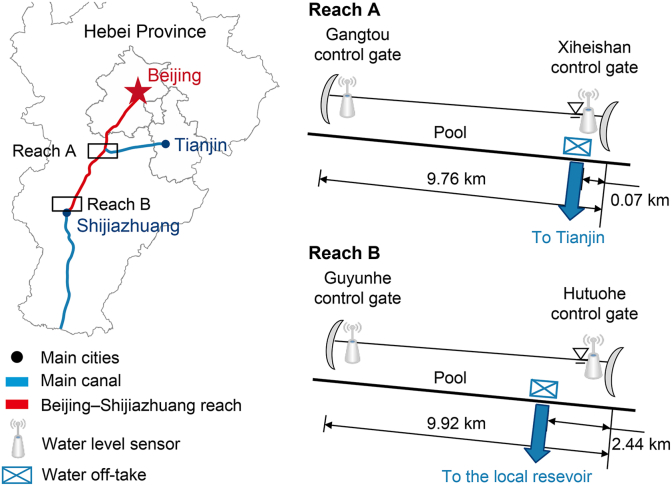
Location and schematic configuration of the two study reaches (Reach A and Reach B) in the Beijing–Shijiazhuang section of the Middle Route of China's South-to-North Water Diversion Project. The figure identifies the upstream and downstream control gates, water-level sensor locations, offtake positions and directions, and key reach lengths.

The proposed models are evaluated using observational data from two canal reaches (Reach A and Reach B) along the Middle Route of the South-to-North Water Diversion Project of China (Fig. 2). These two reaches are located in the Beijing–Shijiazhuang section of the project in Hebei Province, North China, and are representative of regulated reaches within this large canal system. Although both reach the same system, they differ in geometric configuration and operational characteristics and are therefore treated as distinct test cases.

Reach A is characterized by relatively large offtake discharges with modest short-term variability, representing a substantial proportion of the main canal flow. A continuous nine-day dataset collected under typical operating conditions is used for detailed model comparison. In contrast, Reach B is represented by a longer 27-day dataset collected in a different season. This dataset exhibits greater variability, including abrupt changes and pronounced fluctuations associated with non-routine operational events. Additionally, the offtake discharge in Reach B constitutes a smaller proportion of the main canal flow compared to Reach A. The extended dataset is primarily used to evaluate model performance and robustness across a wider range of operating scenarios. The geometric characteristics, flow ranges, and data durations for both reaches are summarized (Table 1).

**Table 1:** Geometric and hydraulic characteristics of Reach A and Reach B.

Reach	Bottom width (m)	Side slope	Bed slope	*Q*_u_ (m^3^ s^−1^)	*Q*_d_ (m^3^ s^−1^)	*H*_u_ (m)	*H*_d_ (m)	*q* (m^3^ s^−1^)
Reach A	19.5	2.5	0.00004	79–88	45–54	3.6–3.9	4.2–4.5	30–35
Reach B	10.0	3.0	0.00004	153–178	130–175	4.8–5.2	5.0–5.4	5–27

Note: *Q*_u,_ future discharge plans at the upstream end; *Q*_d_, future discharge plans at the downstream end; *H*_u_, upstream water level; *H*_d_, downstream water level; *q*, offtake discharge.

Both case studies share several common features: (1) trapezoidal cross-sections with wide and shallow profiles; (2) offtakes located near the downstream end; and (3) mild, nearly uniform bed slopes of 0.00004. Due to the limited sensor availability and monitoring data, this study excludes the effects of evaporation, leakage, and other loss processes in predicting offtake discharge.

### 3.3 Simulation setup and model training

For real-time prediction, the output time step of both the MDN and PgMDN models is set to 20 min, consistent with the data acquisition frequency. The LSTM layers use historical input sequences spanning six time steps (*T* = 6), corresponding to a 2-h window.

We adopted a rolling-horizon prediction scheme, in which fixed-length training, validation, and test sets are sequentially updated to simulate real-time operation. An initial three-day training period is used to initialize the models. For Reach A, this is followed by a six-day rolling prediction period based on the nine-day dataset, using fixed-length training (72 h), validation (12 h, sixfold), and test (24 h) sets. For Reach B, the same approach is applied, resulting in a 24-day rolling prediction period enabled by the extended dataset. Under this framework, geophysical parameters are assumed to remain approximately constant within each analyzed time window.

Both MDN and PgMDN models are trained using the adaptive moment estimation (Adam) optimizer [50]. The learning rate, first-moment decay rate, and second-moment decay rate are set to 0.01, 0.9, and 0.999, respectively, based on established practice and validation results. Hyperparameters for the LSTM layers are determined through grid search within ranges informed by prior studies [51]. Specifically, the number of hidden units is tested at {40, 60, 80, 100}, the number of epochs at {100, 150, 200, 300}, and the L2 regularization weight at {10^−4^, 5 × 10^−4^, 10^−3^, 5 × 10^−3^}.

Hyperparameter selection is conducted using sixfold cross-validation within each 72-h training window, constituting an automated tuning procedure. Due to the relatively high dimensionality of the output, a larger number of hidden units is required to ensure sufficient model capacity. The final configuration includes 80 units per LSTM layer, 200 training epochs, a full batch size of 216 samples, and an L2 regularization weight of 0.001.

All input features are normalized to the range [0,1] prior to training. No early stopping or learning rate decay is applied. These settings are used consistently for both the MDN and PgMDN models. All numerical experiments are conducted in MATLAB R2019b on a Windows system equipped with an Intel Core i7-9700 CPU (3.00 GHz) and 16 GB of random access memory.

## 4 Results

### 4.1 Application of MDN to synthetic data

**Fig. 3:**
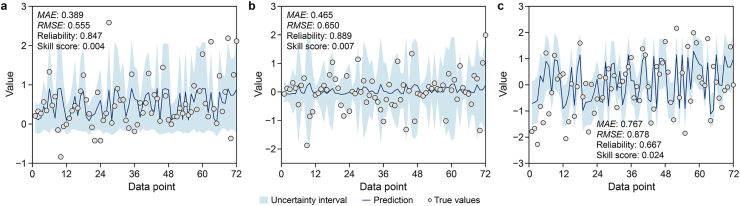
Predictive performance of the MDN model for Case 1 (**a**), Case 2 (**b**), and Case 3 (**c**), together with the associated 90% predictive uncertainty intervals. MDN, mixture density network; *MAE*, mean absolute error; *RMSE*, root mean square error.

The MDN model exhibits distinct predictive performance and associated uncertainty intervals across the three synthetic data patterns (Fig. 3). Across the three cases, the MDN model performs better in Cases 1 and 2, which are characterized by simpler functional relationships, as reflected by higher reliability and skill scores closer to zero. In contrast, Case 3, which represents a more complex nonstationary time series, exhibits higher *MAE* and *RMSE* values along with lower reliability.

These results indicate that while the MDN can capture arbitrary and non-standard distributions, its predictive accuracy and uncertainty calibration degrade as data complexity and temporal variability increase.

Although these synthetic experiments demonstrate the MDN's ability to learn complex conditional distributions, they are conducted under simplified conditions. We therefore next examined the performance of both MDN and PgMDN in real-world systems (Section 4.2).

### 4.2 Comparison of MDN and PgMDN using real system data

#### 4.2.1 Prediction performance on the short-term dataset

Following the training setup described in Section 3.3, this section presents model performance over a continuous six-day prediction period.

**Fig. 4:**
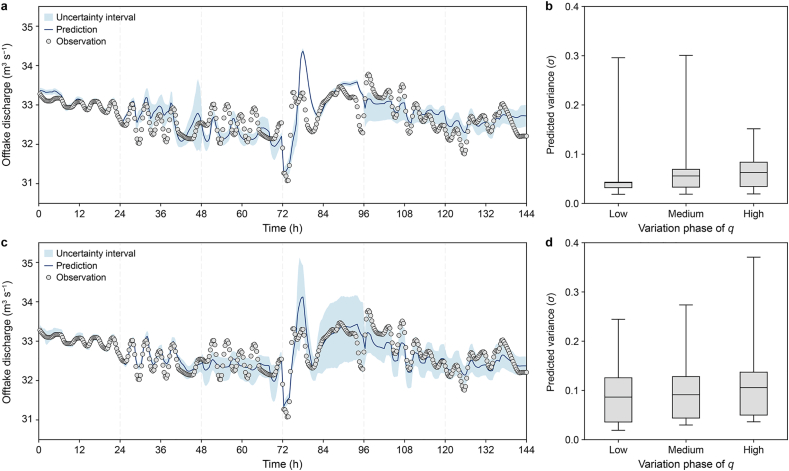
Comparison of MDN and PgMDN predictions for Reach A. **a**,**c**, Time-series predictions from the MDN (**a**) and PgMDN (**c**), shown together with observations and 90% predictive uncertainty intervals across 432 test samples. **b**,**d**, Distributions of the predicted variances (σ) from the MDN (**b**) and PgMDN (**d**) for low, medium, and high variation phases of the offtake discharge (*q*). Variation phases were defined by the rate of change of *q*: low, 0.00–0.06 m^3^ s^−1^ h^−1^ (188 samples); medium, 0.06–0.30 m^3^ s^−1^ h^−1^ (172 samples); and high, 0.30–2.00 m^3^ s^−1^ h^−1^ (72 samples). MDN, mixture density network; PgMDN, physics-guided mixture density network.

The predicted *q* values and their associated uncertainty intervals evolve over time, along with the distribution of predictive *σ* values across different variation phases of *q* (Fig. 4). The PgMDN model more accurately captures short-term fluctuations in *q* than the MDN model, particularly during the 24–48 h and 72–144 h periods. In addition, PgMDN provides more consistent and realistic uncertainty intervals compared to MDN.

For example, MDN produces abrupt, overly narrow uncertainty intervals between 72 h and 96 h, while generating excessively wide intervals during the other periods (Fig. 4a). This behavior is further illustrated in the corresponding box plots. The MDN exhibits relatively concentrated distributions of predicted *σ* values, with no clear relationship to variations in *q*. Notably, it produces the smallest spread in predicted *σ* during periods of high variability (Fig. 4a), indicating limited responsiveness of uncertainty estimates to dynamic system conditions.

In contrast, the PgMDN exhibits progressively wider *σ* distributions as the variability of *q* increases (Fig. 4b). This behavior suggests that the model appropriately adjusts predictive uncertainty in response to dynamic system changes. These results demonstrate that incorporating physical constraints enables the PgMDN to better capture uncertainty under rapidly evolving hydraulic conditions.

**Fig. 5:**
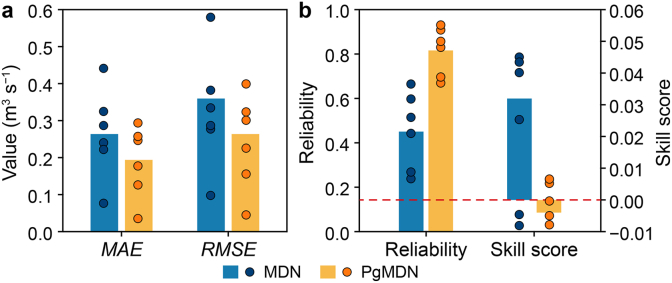
Performance comparison between MDN and PgMDN for Reach A. **a**, Accuracy metrics: *MAE* and *RMSE*. **b**, Uncertainty metrics: reliability and skill score. Bars represent global metrics across the 6-day prediction period (432 samples), while scattered dots represent daily evaluation values (72 samples). The red dashed line indicates the zero reference for the skill score. MDN, mixture density network; PgMDN, physics-guided mixture density network. *MAE*, mean absolute error; *RMSE*, root mean square error.

MDN and PgMDN are compared in terms of both accuracy and uncertainty metrics (Fig. 5). The PgMDN achieves significantly improved prediction accuracy (*MAE* = 0.20, *RMSE* = 0.26; Fig. 5a), reducing the MDN's corresponding errors by 28% and 26%, respectively.

From the perspective of uncertainty quantification, PgMDN also demonstrates substantial improvements. It achieves a higher *R*-index and a lower absolute *S*-index compared to MDN. At the 90% confidence level, reliability increases from 0.45 to 0.82. Furthermore, the negative *S*-index value, which is close to zero, indicates that PgMDN produces more conservative yet well-balanced uncertainty intervals relative to MDN.

The scatter points represent daily performance across six forecasting windows (Fig. 5). PgMDN exhibits less day-to-day variation in *MAE* and *RMSE* than MDN, with even greater stability observed in the *R*-index and *S*-index. Overall, consistent improvements across all evaluation metrics demonstrate that PgMDN outperforms MDN in terms of accuracy, reliability, and robustness.

#### 4.2.2 Performance validation under extended conditions

**Fig. 6:**
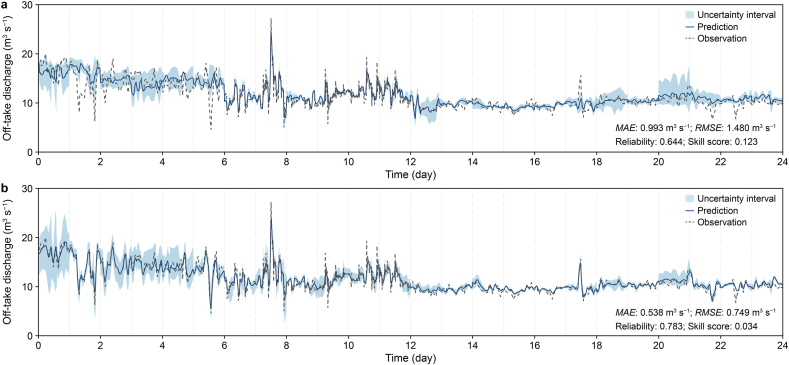
Comparison of MDN and PgMDN predictions for Reach B over the 24-day test period. Predictions from the MDN (**a**) and PgMDN (**b**) compared with observations, with 90% predictive uncertainty intervals, over the 24-day test period (1728 samples). MDN, mixture density network; PgMDN, physics-guided mixture density network. *MAE*, mean absolute error; *RMSE*, root mean square error.

The prediction performance of MDN and PgMDN over the 24-day forecasting period in Reach B is compared (Fig. 6). The PgMDN achieves substantially lower prediction errors, reducing *MAE* and *RMSE* from 0.99 and 1.48 m^3^ s^−1^ (MDN) to 0.54 and 0.75 m^3^ s^−1^, respectively. In addition, PgMDN provides more reliable uncertainty estimates, increasing reliability from 0.64 to 0.78 and reducing the skill score from 0.12 to 0.03. The predicted uncertainty intervals produced by PgMDN adapt more effectively to abrupt fluctuations, particularly during periods of significant operational change. This indicates improved stability and more accurate characterization of uncertainty over extended time horizons.

**Fig. 7:**
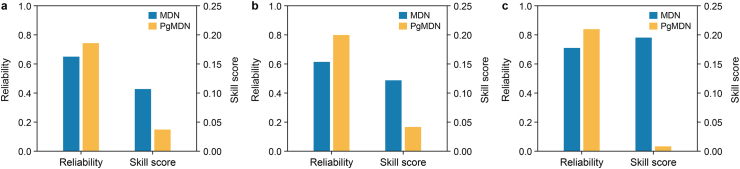
Comparison of MDN and PgMDN performance in Reach B across different variation phases of the offtake discharge (*q*). Uncertainty metrics (reliability and skill score) for low (**a**), medium (**b**), and high (**c**) variation phases. Variation phases were defined by the rate of change of *q*: low, 0.0–0.2 m^3^ s^−1^ h^−1^ (864 samples); medium, 0.2–1.0 m^3^ s^−1^ h^−1^ (604 samples); and high, 1.0–9.0 m^3^ s^−1^ h^−1^ (260 samples). MDN, mixture density network; PgMDN, physics-guided mixture density network.

To further assess model robustness under varying operating conditions, the 24-day dataset is categorized into low, medium, and high variability levels based on the rate of change in offtake discharge. PgMDN consistently achieves higher reliability and improved uncertainty performance than MDN across all variability levels (Fig. 7). Specifically, PgMDN achieves reliability values of approximately 0.74 under low-level variability and up to 0.84 under high variability. In contrast, MDN exhibits lower and more variable coverage across different conditions. In addition, PgMDN produces skill scores closer to zero, with the best performance observed under high-variability conditions. These findings demonstrate that the proposed PgMDN model improves uncertainty quantification under both stable and highly dynamic operating conditions.

#### 4.2.3 Model generalization ability

We conducted two tests to evaluate model generalization. In the first test, both MDN and PgMDN are trained on data from Reach A during Day 1 (0–24 h), which represent relatively stable conditions, and then tested on Day 4 (72–96 h), characterized by sharp fluctuations. In the second test, both models are trained on data from Days 3–5 in Reach B under typical regular conditions and evaluated on Day 8, which exhibits pronounced variability and abrupt changes. These tests assess the robustness of PgMDN relative to MDN under unfamiliar and rapidly changing operational dynamics.

**Fig. 8:**
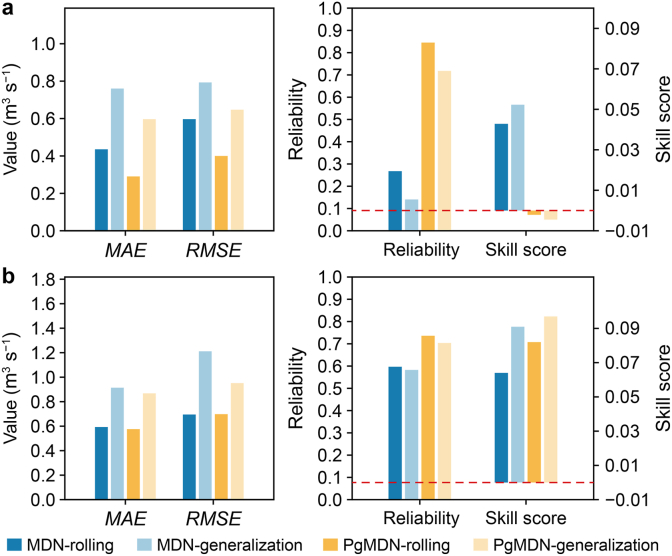
**Comparison of MDN and PgMDN performance under rolling and generalization tests in Reach A (a) and Reach B (b).** The red dashed line indicates the zero reference for the skill score. MDN, mixture density network; PgMDN, physics-guided mixture density network. *MAE*, mean absolute error; *RMSE*, root mean square error.

Prediction errors increase for both models under generalization scenarios (Fig. 8), reflecting the increased difficulty of forecasting under unseen conditions. This effect is more pronounced for Reach B, which exhibits more frequent and intense fluctuations, making it a more challenging test case.

Despite these challenges, PgMDN consistently achieves lower *MAE* and *RMSE* than MDN across both reaches and test scenarios. In terms of uncertainty quantification, PgMDN maintains stable reliability and skill score values. Even under highly dynamic conditions in Reach B, PgMDN preserves reliability levels of approximately 0.7–0.8 and skill scores close to zero, whereas MDN exhibits more pronounced degradation. These results indicate that incorporating physics-guided constraints enhances model robustness and generalization, particularly under abrupt, previously unseen operating conditions.

### 4.3 Analysis of input contributions

**Fig. 9:**
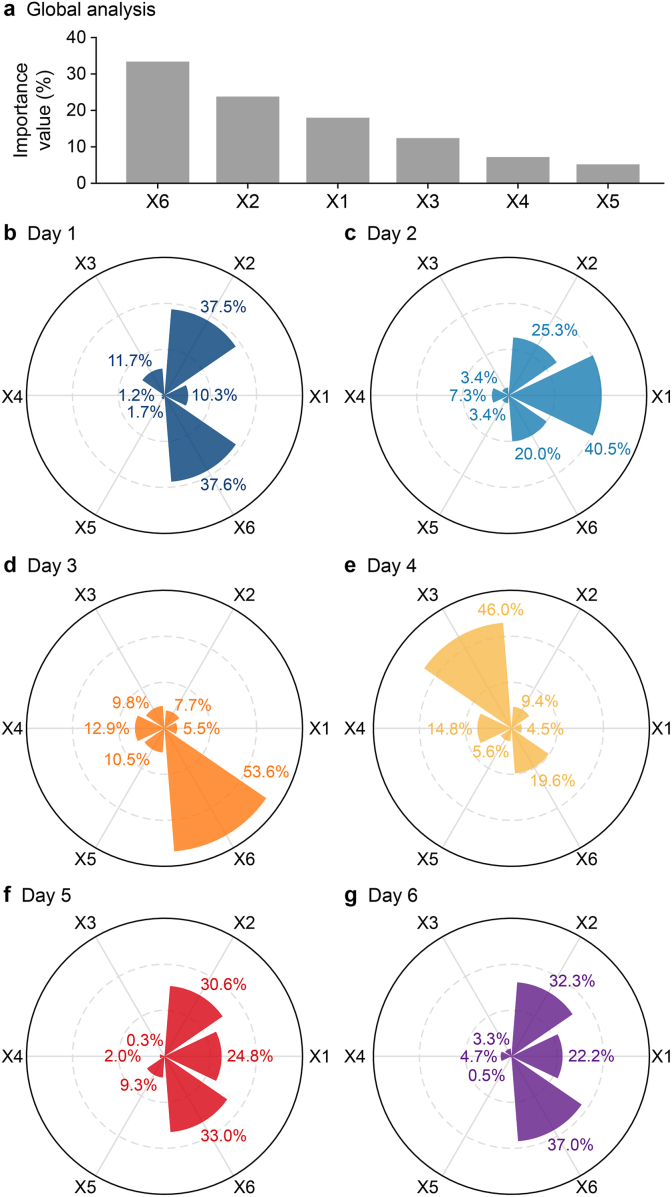
**Global and day-specific importance of input variables over the 6-day prediction period. a**, Global importance ranking based on all 432 samples. **b**, Day-specific importance distributions for days 1–6, based on 72 samples per day. Input variables are defined as *X*1, Δ*H*_u_, upstream water-level variation; *X*2, Δ*H*_d_, downstream water-level variation; *X*3, *Q*_u_, upstream discharge; *X*4, *Q*_d_, downstream discharge; *X*5, *H*_d_, downstream water level; *X*6, *H*_d_Δ*H*_d_, interaction term between downstream water level and downstream water-level variation.

Shapley values are computed for each test data point to quantify the contribution of input variables to predictive uncertainty, and these values are averaged daily. To facilitate comparison, the average Shapley values are expressed as percentages, indicating the relative contribution of each variable on each day for Reach A (Fig. 9), with overall contributions across the six-day period summarized in panel a and daily contributions in panels b–g.

The global results (Fig. 9a) indicate that variables related to water-level variation—namely, *X*1, *X*2, and *X*6—are the primary drivers of predictive uncertainty. Daily results (Fig. 9b–g) further show that a nonlinear term *X*6 (*H*_d_Δ*H*_d_) plays a dominant role on most days, contributing between 19.6% and 53.6%.

Additionally, *X*1 (Δ*H*_u_) and *X*2 (Δ*H*_d_) generally exert greater influence on uncertainty than *X*3 (*Q*_u_) and *X*4 (*Q*_d_), particularly on Days 2, 5, and 6, where their contributions exceed 20% (Fig. 9c–f,g). However, this pattern varies across days. For example, on Day 3, only *X*6 exhibits a significant contribution, while the influence of other variables is minimal (Fig. 9d). This may help explain the reduced model performance observed on Day 3 (Fig. 4), where the model struggles to capture the trend of actual offtake discharge.

On Day 4, *X3* (*Q*_u_) becomes the dominant contributor, accounting for 46% of total importance, indicating that inflow discharge plays a key role in driving uncertainty during periods of rapid change. In contrast, boundary discharge variables (*X*3 and *X*4) have relatively minor contributions under stable conditions, typically below 12%. Meanwhile, *X*5 (*H*_d_) shows a consistently small but highly variable contribution across all days, ranging from 0.5% to 10.5% (Fig. 9e).

Overall, these results suggest that predictive uncertainty is primarily driven by variables related to hydraulic change (*X*1 and *X*2) and the nonlinear downstream response (*X*6). However, under rapidly changing conditions, inflow discharge (*X*3) becomes a more dominant factor influencing uncertainty.

The SHAP results indicate that dominant drivers of predictive uncertainty vary with operating conditions. Specifically, *X*1 and *X*2 exceed 20% on Days 2, 5, and 6, whereas *X*3 accounts for 46% of total importance on Day 4, enabling operators to adjust their monitoring focus accordingly. During periods of pronounced variability in offtake discharge, inflow discharge becomes a key variable for operational monitoring and decision support. Under relatively stable conditions, variations in downstream water levels provide more relevant information for routine system management.

## 5 Discussion

### 5.1 Sensitivity and robustness analysis

To evaluate PgMDN's sensitivity to the weights of the loss terms, we conducted a comparative analysis using the nine-day dataset from Reach A. The data loss weight is fixed at *λ*_d_ = 1, while the two physics-related terms are varied around their default values. Five configurations of are considered: (*λ*_d_, *λ*_p_, *λ*_c_) = (1, 1, 1), (1, 0.5, 1), (1, 1, 0.5), (1, 5, 1), and (1, 1, 5). All other training settings are kept identical.

**Fig. 10:**
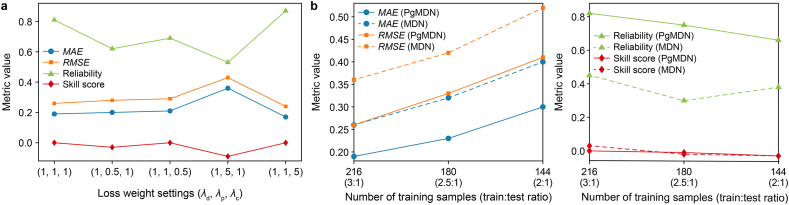
**Robustness analysis of PgMDN under different loss-weight settings and training sample sizes. a**, PgMDN performance under different combinations of *λ*_d_, *λ*_p_, and *λ*_c_. **b**–**g**, Comparison of MDN and PgMDN performance under different training sample sizes at Day 1 (**b**), Day 2 (**c**), Day 3 (**d**), Day 4 (**e**), Day 5 (**f**), and Day 6 (**g**). MDN, mixture density network; PgMDN, physics-guided mixture density network. *MAE*, mean absolute error; *RMSE*, root mean square error; *λ*_d_, *λ*_p_, and *λ*_c_, the weighting coefficients of the data NLL term, the physical NLL term, and the physics-guided term, respectively; NLL, negative log-likelihood.

The performance of PgMDN under these configurations is presented (Fig. 10a). *MAE* and *RMSE* remain relatively stable under moderate perturbations of the physics-related weights, indicating that the model is not overly sensitive to their selection. However, excessively increasing the physical NLL term (1, 5, 1) degrades both accuracy and uncertainty performance, suggesting that overly strong physical constraints may restrict model flexibility and limit the learning of data patterns. In contrast, increasing the weight of the physics-guided term (1, 1, 5) improves both accuracy and reliability, underscoring its greater benefit. Overall, PgMDN demonstrates robust performance and does not depend on a narrowly tuned weighting configuration.

To further assess robustness under data-scarce conditions, the forecasting horizon is fixed at 72 samples, while the amount of training data is progressively reduced. For each training size, MDN and PgMDN are trained and evaluated on the same test period under identical experimental settings.

Both models show degraded performance as the training data decrease, as reflected by increasing *MAE* and *RMSE* and declining reliability (Fig. 10b). However, PgMDN consistently achieves lower prediction errors and substantially higher reliability than MDN across all data sizes. Moreover, the degradation in PgMDN performance is more gradual, whereas MDN exhibits a sharper decline when training data becomes limited. The skill score of PgMDN remains close to zero under different data sizes, indicating stable probabilistic behavior, while MDN shows larger deviations under small-sample conditions.

### 5.2 Comparison with previous studies

As discussed in the Introduction, UQ in canal prediction has been approached through ensemble hydrodynamic models, stochastic operational frameworks, and data-driven probabilistic methods. Physics-based methods ensure physical consistency but often incur high computational costs, whereas purely data-driven models typically rely on scenario perturbations and may lack robustness under changing conditions.

In contrast, PgMDN models uncertainty by directly learning the conditional probability distribution of canal states, allowing it to capture complex, input-dependent uncertainty arising from unobserved operational disturbances without relying on predefined scenarios. Physical knowledge is incorporated into this probabilistic framework to constrain the learned distributions, ensuring consistency with fundamental hydraulic principles.

The superior accuracy and reliability of PgMDN predictions can be attributed to two primary factors. First, the MDN architecture with LSTM layers effectively captures implicit multimodal patterns in the data [38,39] (Fig. 3). Second, incorporating physical constraints enhances sensitivity to hydrodynamic laws, leading to more consistent predictions. This effect is reflected in the improved performance metrics (Fig. 6, Fig. 9) and aligns with findings from previous work [41].

Furthermore, the SHAP analysis (Fig. 8) suggests that the model captures physically meaningful relationships. Uncertainty is primarily driven by water-level variations under normal conditions, whereas inflow variations dominate during periods of rapid operational change. This behavior is consistent with the established understanding of canal system dynamics, supporting the interpretability of the model [52].

Compared to prior applications of MDN models [27,37,38], the proposed PgMDN demonstrates improved performance on small and real-world datasets by embedding physical constraints directly into the loss function. For variables such as offtake discharge, which are governed by short-term hydrodynamic processes rather than long-term hydrologic trends [38,39], PgMDN provides reliable and robust multimodal predictions.

This physics-guided framework enables the model to generate physically consistent, state-dependent conditional distributions while preserving the flexibility of deep learning. Such capability is particularly valuable for canal system operations, especially under short-horizon monitoring conditions. The resulting probabilistic forecasts of offtake discharge can be integrated into hydrodynamic models to estimate potential water level ranges under varying scenarios [8].

Accordingly, the PgMDN model is well-suited for real-time canal management, where predictive intervals can support adaptive scheduling and risk-aware control [53]. These probabilistic outputs enable more informed operational decisions, such as adjusting safety margins or optimizing the timing of control actions, rather than relying solely on deterministic forecasts. In this sense, PgMDN provides a practical framework for bridging physical hydrodynamic modeling, data-driven UQ, and operational decision support.

### 5.3 Limitations and future work

Despite its demonstrated performance, several limitations remain. First, the mass balance formulation relies on inflow and outflow discharges derived from a simplified 1D hydraulic model, which may not fully capture complex hydraulic interactions in real canal systems. As a result, local deviations from strict mass balance may occur under certain operating conditions, particularly when additional physical uncertainties are present.

In this study, such complexities are implicitly accounted for through real-time water level observations, which serve as key inputs for offtake discharge prediction. While this assumption is reasonable in practice, it remains an approximation. The multi-term loss formulation introduces an inherent trade-off between local physical consistency and data-driven flexibility. Because the physical constraint is applied locally, residual discrepancies may be absorbed by the data likelihood term. Further improvements could involve refining the hydraulic model or incorporating additional observational data.

Second, the PgMDN employs relatively soft physical constraints compared to physics-informed neural networks [54,55]. While this enhances flexibility, it may reduce stability under certain input conditions. Incorporating stronger or more explicit physical constraints may further improve the model's ability to capture hydrodynamic behavior.

Additionally, although relatively long datasets are used in this study, the evaluation does not cover a full seasonal cycle. Future work could also extend the framework to multi-step forecasting, enabling better representation of long-term temporal dependencies and seasonal dynamics. Over longer horizons, changes in environmental and geophysical conditions may become increasingly important. Moreover, model validation may be influenced by measurement noise, simplified physical assumptions, and limited temporal coverage, which can introduce variability in performance metrics under certain conditions.

Finally, the current approach relies on a predefined set of hydraulic input features, which may not fully capture higher-order interactions within the system. Future research could explore end-to-end learning approaches, such as attention-based architectures, to implicitly encode physical relationships [56]. Such methods may also help improve the identification of high-risk conditions while maintaining interpretability and real-time applicability. Additionally, broader comparisons with alternative UQ methods remain an important direction for future work.

## 6 Conclusion

This study proposes a novel PgMDN model that integrates physical constraints into a data-driven probabilistic framework to quantify uncertainty in water-offtake discharge predictions. By embedding both a local mass-balance constraint and a coupling between system-state variation and uncertainty into the loss function, the model produces more robust and physically consistent conditional distributions.

The baseline MDN is first validated using small-sample synthetic datasets. The proposed PgMDN model is then evaluated using real-world canal system data across multiple time scales and operating conditions. Compared to the standard MDN, PgMDN demonstrates consistently superior performance in both point prediction and uncertainty quantification. The main findings are as follows.

(1) Compared to the standard MDN, which performs well on synthetic datasets, PgMDN achieves more accurate predictions on small real-world datasets, reducing *MAE* and *RMSE* by 28% and 26%, respectively.
(2) PgMDN improves prediction reliability from 0.45 to 0.82 and provides more conservative yet well-balanced uncertainty estimates while maintaining stable generalization under transfer testing and reduced training data conditions.
(3) SHAP analysis shows predictive uncertainty is primarily driven by water level variations under normal conditions and by inflow discharge during rapid changes, supporting the physical interpretability of the model.

In summary, this study demonstrates that incorporating physical knowledge into neural network architectures can significantly enhance their robustness, generalization capability, and interpretability. This approach provides a promising pathway for integrating data-driven modeling with physical understanding, enabling more resilient and adaptive management of water resources and environmental systems.

## CRediT authorship contribution statement

**Wangjiayi Liu:** Writing – original draft, Visualization, Methodology, Investigation, Formal analysis, Conceptualization. **Guanghua Guan:** Writing – review & editing, Supervision, Software, Funding acquisition, Conceptualization. **Xiaonan Chen:** Resources, Data curation. **Liangsheng Shi:** Validation, Project administration. **Guangtao Fu:** Writing – review & editing, Supervision, Conceptualization. **Dragan Savic:** Writing – review & editing, Supervision, Conceptualization.

## Declaration of competing interest

The authors declare that they have no known competing financial interests or personal relationships that could have appeared to influence the work reported in this paper.

## Data Availability

All data, models, or codes that support the findings of this study are available from the corresponding author upon reasonable request.
